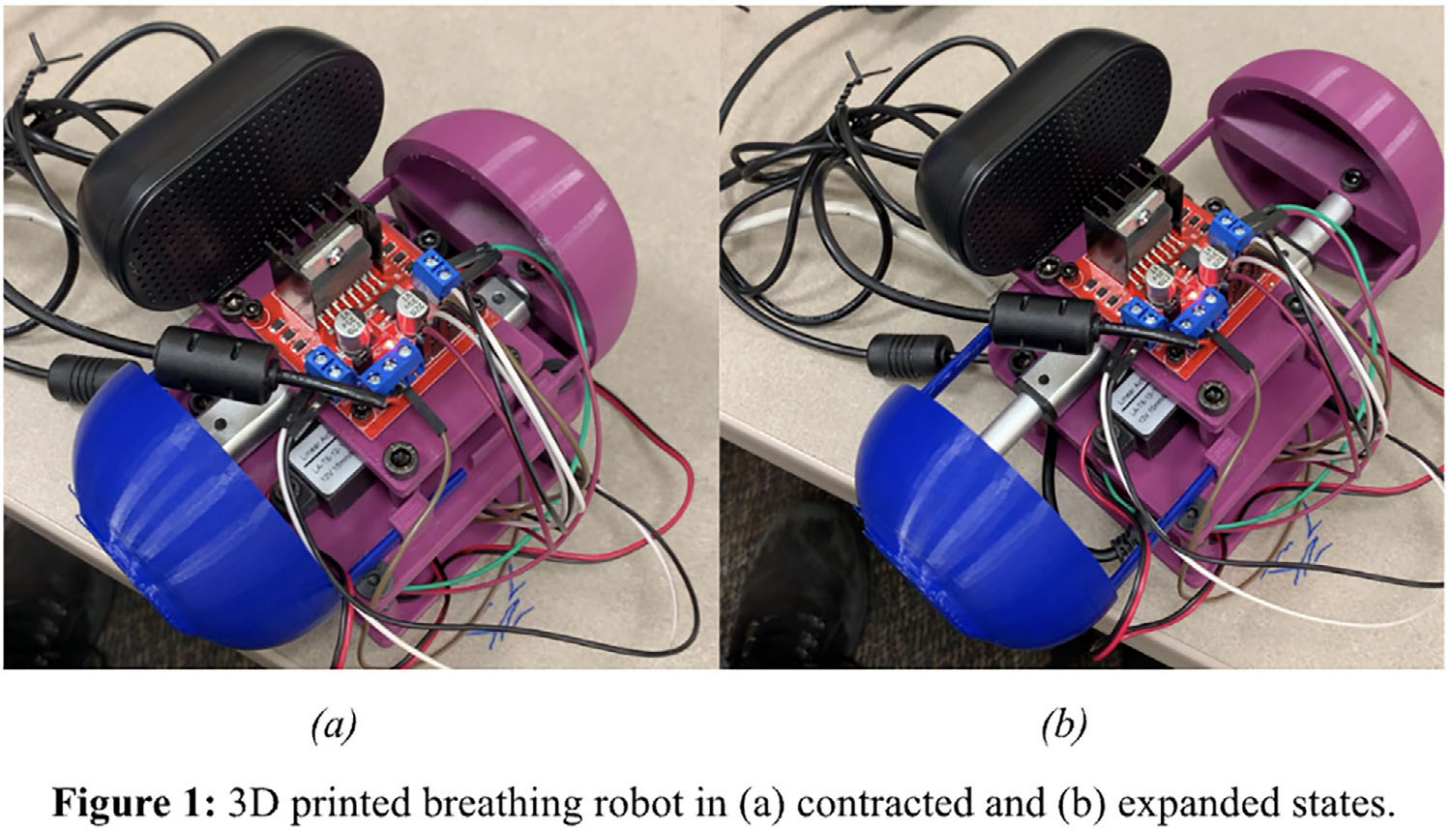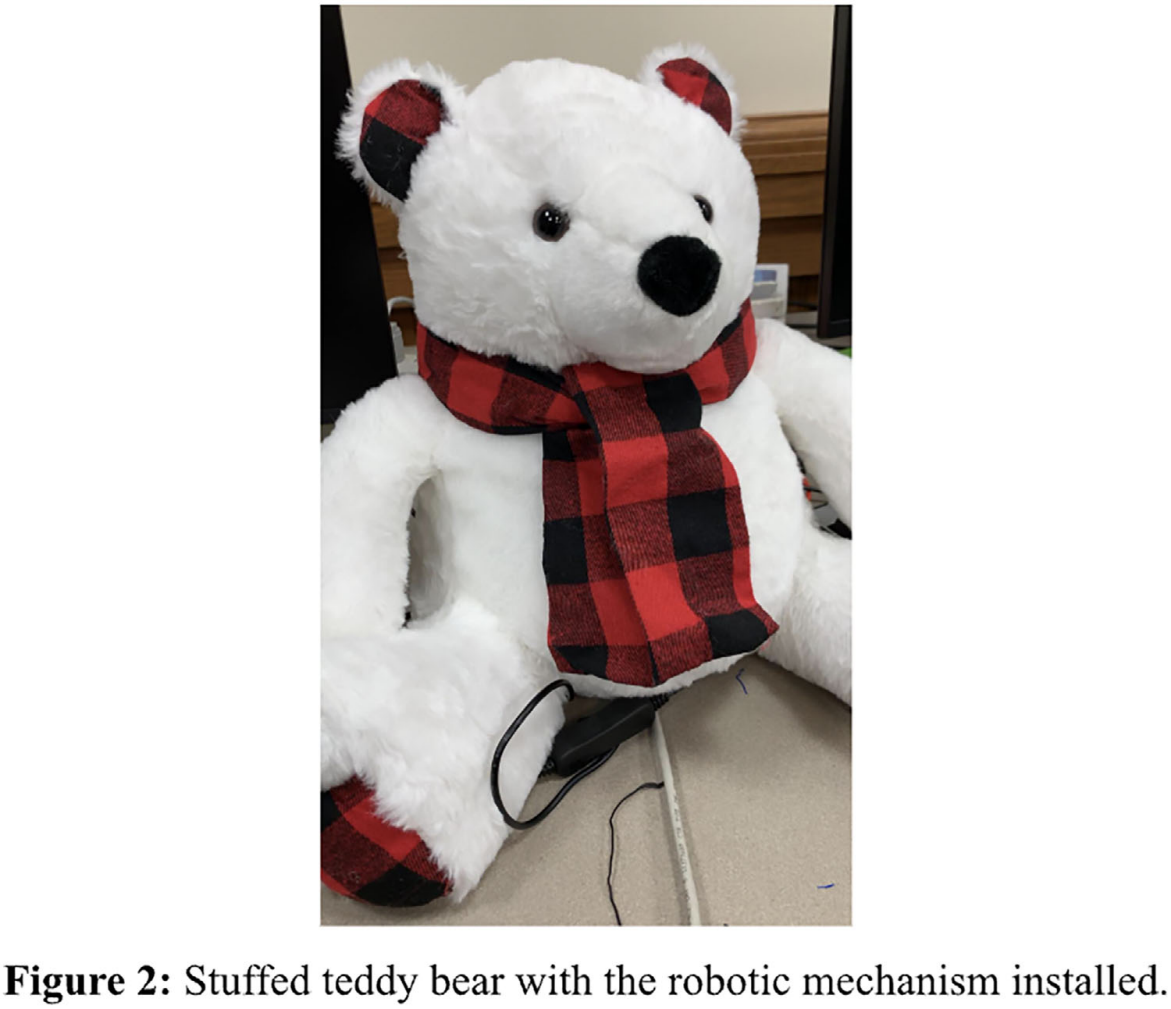# BreatheBot Buddy: Fostering Cognitive Well‐being with a Cuddly Companion for Regulated Breathing

**DOI:** 10.1002/alz.086716

**Published:** 2025-01-09

**Authors:** Arshia A Khan, Matthew Sawchuk, Sabir Saheel, Jack Quigley, Detlef Heck

**Affiliations:** ^1^ University of Minnesota Duluth, Duluth, MN USA

## Abstract

**Background:**

Regulated breathing is increasingly recognized as a vital component in enhancing cognition. Scientific studies suggest that intentional and controlled breathing techniques, such as deep and rhythmic breathing, can promote relaxation, reduce stress, and improve oxygen flow to the brain [1, 2]. Consequently, this may contribute to heightened cognitive function, better concentration, and increased mental clarity, accentuating the importance of incorporating mindful breathing practices as a potential avenue for cognitive improvement [3].

The increasing recognition of the cognitive benefits of regulated breathing is leading to the development of innovative technologies, such as robots, for better breathing practices. These technologies can be used to potentially enhance the cognitive well‐being and mindfulness of people in their daily routines.

**Methods:**

In order to accomplish a breathing robot, the entire design consists of two linear actuators controlled by an L298N Motor Driver Interface and a Raspberry Pi 3B. The Raspberry Pi coordinates both linear actuators to extend, hold, and contract in opposite directions for 4 seconds at each stage. Additionally, the mechanical design utilizes two “push caps” that push at the sides of a contained space, such as a stuffed animal, simulating breathing (see Fig. 1). This design simulates a breathing pattern in any stuffed animal (see Fig. 2).

**Results:**

The developed robot underwent rigorous testing in both a lab and poster presentation setting. Various case studies were examined during the poster presentation, revealing the effectiveness of a tool designed to aid in regulated breathing. The present robot demonstrated increased participant engagement in mindful breathing practices, which suggests a promising avenue for leveraging robot‐assisted therapies to enhance cognitive well‐being in people.

**Conclusion:**

The present project aimed to build a robot that fosters cognitive well‐being through regulated breathing. The case study findings of this project highlight the effectiveness of a cuddly bear‐shaped robot as a valuable tool for promoting regulated breathing, offering positive implications for integrating such robotic assistance in enhancing cognitive well‐being.